# Dissecting Cellular Heterogeneity Based on Network Denoising of scRNA-seq Using Local Scaling Self-Diffusion

**DOI:** 10.3389/fgene.2021.811043

**Published:** 2022-01-10

**Authors:** Xin Duan, Wei Wang, Minghui Tang, Feng Gao, Xudong Lin

**Affiliations:** ^1^ Guangdong Provincial Key Laboratory of Sensor Technology and Biomedical Instrument, School of Biomedical Engineering, Sun Yat-Sen University, Guangzhou, China; ^2^ Biomedical Big Data Center, Department of Gynecology, Huzhou Maternity and Child Health Care Hospital, Huzhou, China; ^3^ Department of Colorectal Surgery, The Sixth Affiliated Hospital, Sun Yat-sen University, Guangzhou, China; ^4^ Guangdong Provincial Key Laboratory of Colorectal and Pelvic Floor Diseases, Guangdong Institute of Gastroenterology, Supported by National Key Clinical Discipline, Guangzhou, China

**Keywords:** cellular heterogeneity, local scaling affinity, self-diffusion, single-cell clustering, network denoising

## Abstract

Identifying the phenotypes and interactions of various cells is the primary objective in cellular heterogeneity dissection. A key step of this methodology is to perform unsupervised clustering, which, however, often suffers challenges of the high level of noise, as well as redundant information. To overcome the limitations, we proposed self-diffusion on local scaling affinity (LSSD) to enhance cell similarities’ metric learning for dissecting cellular heterogeneity. Local scaling infers the self-tuning of cell-to-cell distances that are used to construct cell affinity. Our approach implements the self-diffusion process by propagating the affinity matrices to further improve the cell similarities for the downstream clustering analysis. To demonstrate the effectiveness and usefulness, we applied LSSD on two simulated and four real scRNA-seq datasets. Comparing with other single-cell clustering methods, our approach demonstrates much better clustering performance, and cell types identified on colorectal tumors reveal strongly biological interpretability.

## Introduction

The cells are the fundamental structural unit in biological systems. For centuries, biologists have discovered that multicellular biological tissues are characterized by different cell types and can be distinguished according to their size and shape. Many studies have confirmed that genome-wide mRNA expression obtained from cell populations exhibits potential value in biological analysis (Bacher and Kendziorski, 2016; Guo et al., 2018). Traditional microarrays, whole-genome RNA-seq sequencing, obtain the average value of tens of thousands of gene expressions from bulk-tissue samples. Although this sequencing technology is applied in many areas (Xu et al., 2019; Chen et al., 2020), it cannot measure the gene expression value in a single cell. In recent years, single-cell RNA sequencing (scRNA-seq) technologies have been developed as an attractive tool to reveal cell functional diversity and heterogeneity, bringing new insights into the biological systems (Pelkmans, 2012; Kaur et al., 2019). The rapid development of the scRNA-seq technique has enabled the dramatic increase of single-cell transcriptome data, which bring opportunities and challenges to the computational biology approaches (Pelkmans, 2012; Buettner et al., 2015; Kaur et al., 2019). In a single-cell heterogeneity study, unsupervised clustering of transcriptomes profiled by scRNA-seq is an essential intermediate step to identify cell types, followed by analyzing cell biological mechanisms (Luecken and Theis, 2019). Single-cell clustering analysis has the purpose to explain cellular heterogeneity based on the categorization of cells into groups, which exhibit similar gene expression levels. However, scRNA-seq data are so sparse with high dimensionality, plentiful zero count observations, as well as transcript amplification noise. In addition, scRNA-seq displays a high variability in gene expression levels, which further complicates the clustering issue. The widely used clustering algorithms for bulk RNA-seq, such as K-means (Aggarwal and Reddy, 2018), hierarchical clustering (Herrero et al., 2001), non-negative matrix factorization (NMF) (Wang et al., 2021), are not effective enough to address the underlying computational and statistical challenges for scRNA-seq. Several single-cell clustering approaches have been developed recently, for instance, SIMLR (Wang et al., 2017) learns a robust cell similarity metric that best fits the data structure *via* combining multiple kernels. SIMLR is scalable and can largely increase clustering performance, but is very time-consuming and requires many memories. Park et al.(Park and Zhao, 2018) proposed a sparse structure by L1 penalty to deal with the sparsity of scRNA-seq. Tian et al.(Tian et al., 2019) proposed scDeepCluster, a deep learning–based method which learns feature representation and clustering by explicit modeling. SC3 (Kiselev et al., 2017) combines multiple clustering solutions through a consensus approach to achieve high accuracy and robustness. CIDR (Lin et al., 2017) approach alleviates the impact of dropouts in scRNA-seq data by incorporating a simple implicit imputation method. Stuart et al. (Stuart et al., 2019) (Hao et al., 2021) developed an R package “Seurat” for analysis and exploration of single-cell RNA-seq data. The “Seurat” package can not only identify and interpret cellular heterogeneity but also allow integrating diverse types of single-cell measurements across different modalities. Zou et al. (2021) presented a fast hierarchical graph-based clustering (HGC) method to construct dendrograms of cells with linear time complexity. Zhu et al. (2019) proposed semisoft clustering with pure cells (SOUP) to classify pure and transitional cells from their profiles. SAME clustering is a mixture model–based approach which aggregates various clustering methods *via* the mixture model ensemble to produce an improved ensemble solution (Huh et al., 2020). The effectiveness of those single-cell clustering methods may decrease due to the low single-cell quality, biological differences, and the measurement dropouts. The reason is that partitioning the cells into different groups usually relies on distance measurement of their gene expression profiles. The popular similarity measurement such as Cosine and Euclidean cannot generalize well across the biological differences and sample noises. In addition, scRNA-seq data in high-dimensional space tend to be sparse, and the efficiency of common distance measurement methods will be greatly reduced. Therefore, the instability of measurement distance brings great challenges to unsupervised clustering algorithms. To overcome the limitation and attempt to obtain an appealing cell affinity, we propose self-diffusion on local scaling affinity (LSSD) to facilitate the similarity metric learning of single-cell RNA-seq for dissecting cell heterogeneity. Our approach belongs to manifold learning which focuses on discovering the underlying embedding representation with an enhanced distance notion (Roweis and Saul, 2000). The local scaling affinity constructs similarity in the space of cells rather than gene measurements with a distance notion. The self-diffusion process allows the derived distances to follow the intrinsic data manifolds.

**FIGURE 1:**
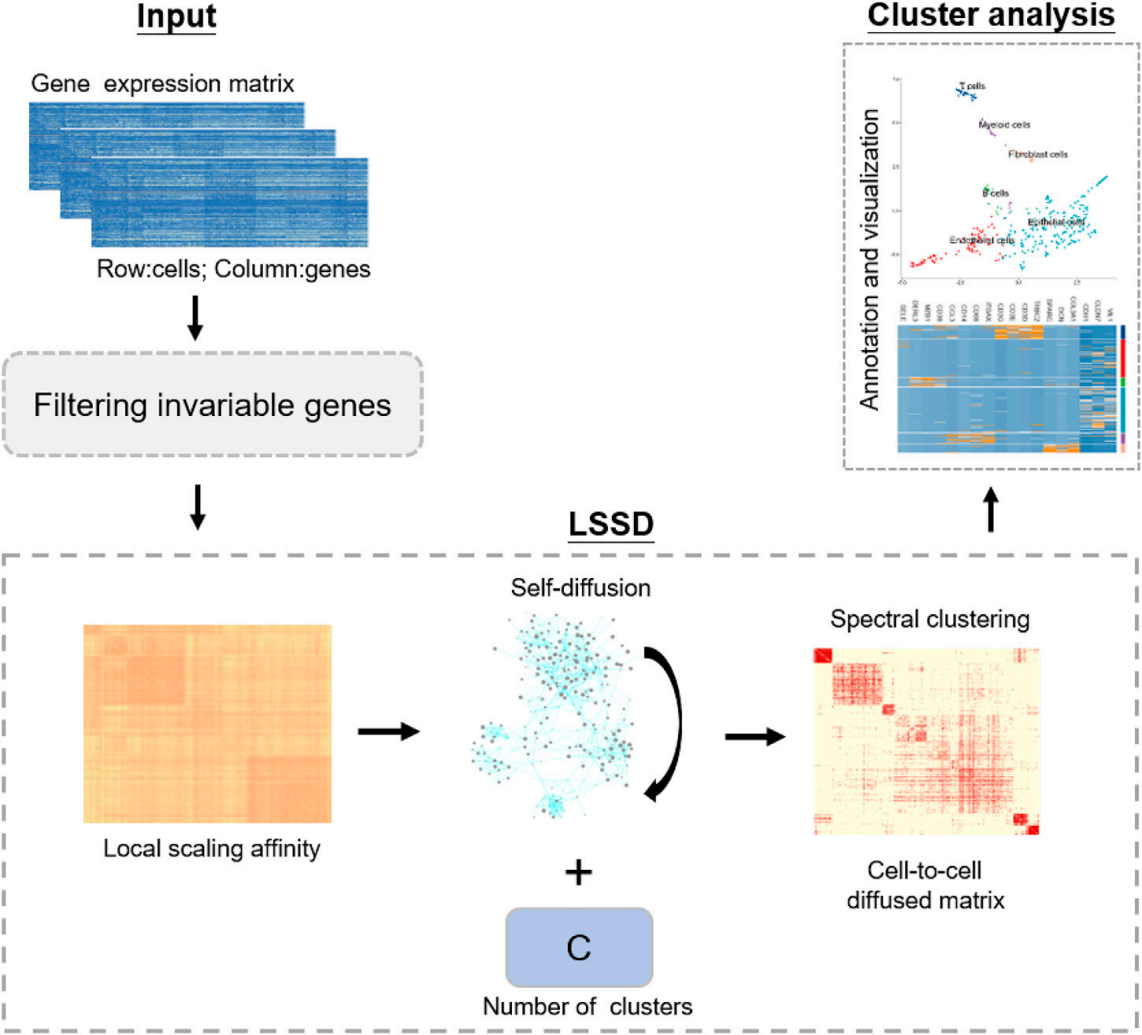
Schematic of our approach, LSSD workflow. LSSD performs self-diffusion on the local scaling affinity constructed by scRNA-seq data. The diffused graph enables effective clustering with C separable cell populations, followed by cell type annotation and visualization.

Our LSSD method applies a stochastic diffusion process on the local scaling affinity, enabling the local similarities to be propagated along the data manifold. Diffusion-based approaches define an average operator by assembling and accumulating all the paths between samples. Among the diffusion-based metric learning, diffusion maps (Haghverdi et al., 2015) first construct sample-to-sample similarity distances and then implement a diffusion process to improve the similarity of input data. The diffusion map takes the advantages of obtaining a global similarity metric notion and implementing multi-scale data analysis from a more natural way by iteratively updating the diffusion steps. Jiang et al. (Jiang et al., 2011) introduced a self-smoothing operator (SSO) which is a diffusion-based approach to improve input similarity metrics and is distinct in that it directly improves the similarity metric using a self-induced smoothing kernel. Self-diffusion was initially proposed by Wang et al. (Jiang et al., 2011; Wang and Tu, 2012) with application for image segmentation and clustering. They then applied a diffusion process to improve the similarity measurement derived from multi-kernel learning for single-cell RNA-seq visualization and analysis (Wang et al., 2017). Self-diffusion belongs to diffusion-based approaches and relies on the assumption that long-range similarities can be calculated by the accumulation of local similarities. Instead of using the Gaussian kernel which suffers from high sensitivity to the hyper-parameters, we implement self-diffusion on local scaling affinity with a new way of performing the diffusion process for dissecting cellular heterogeneity. Local scaling infers the self-tuning of cell-to-cell distances and can eliminate the scale differences, resulting in higher affinities within clusters. The diffusion process can enhance weak measurements of cell-to-cell distance, therefore further facilitating the intercluster cells’ similarity learning and addressing the challenge of noise in scRNA-seq data for the downstream cell identification. LSSD performs similarity learning in the space of cells rather than gene measurements without constructing new embedding spaces. LSSD’s simplicity and efficiency make it an appealing approach for unsupervised clustering analysis of scRNA-seq data. Our approach includes three main phases: (1) constructing local scaling affinity to measure cell similarities on scRNA-seq data, (2) performing self-diffusion process to enhance the cell-to-cell similarities learning, and (3) identifying cell types by clustering on the diffusion map and annotating clusters with known gene markers (Figure 1).

## Materials and Methods

### Data Collection

In this study, we applied LSSD to two simulated datasets and four real scRNA-seq datasets to evaluate its performance. The simulated scRNA-seq data contain 10000 cells, 10000 features with 10 cell groups. The Pollen dataset (Pollen et al., 2014) and Deng dataset (Deng et al., 2014) include 301, 268 cells with 23730, 22431 features, and form 11, 6 cell populations, respectively. The 10X PBMC dataset contains 4271 cells, 16449 features with eight groups (Zheng et al., 2017). The colorectal tumor dataset has 375 tumor cells with 55186 features (Li et al., 2017).

### Self-Diffusion on Local Scaling Affinity

Given a graph G=(V,E) where vertices $V=\left\{x_{1},x_{2}\cdots x_{n}\right\}$ represent n cells, edges *E* are measured by n×n distance matrix **W**. Here, **W** is constructed by using the local scaling method (Zelnik-manor and Perona, 2005), which can alleviate the sensitivity to the hyper-parameters and data scale differences. The affinity distance is defined as follows:

$$W\left(i,j\right)=\exp\left\{\frac{-d^{2}\left(x_{i},x_{j}\right)}{\sigma_{i}\sigma_{j}}\right\},$$ (1)

where $\sigma_{i}$ includes local scaling parameters for each cell $x_{i}$ and $d\left(x_{i},x_{j}\right)$ is the cell-to-cell Euclidean distance. The distance between $x_{i}$ and $x_{j}$ as “seen” by $x_{i}$ is $d\left(x_{i},x_{j}\right)/\sigma_{i}$ , while the converse is $d\left(x_{j},x_{x}\right)/\sigma_{j}$ . This assumption allows the self-tuning of cell-to-cell similarity surrounding cell *i* and *j*. The local scale $\sigma_{i}$ can be defined as $d\left(x_{i},x_{k}\right)$ ; here, $x_{k}$ is the $K^{\prime }$ th neighbor around cell $x_{i}$ . The local scaling distance automatically calculates the scale in samples, addressing the challenge of scaling difference which is problematic for other distance methods. The selection of *K* is independent of the scale. In our analysis, we set *K* = 5, which gives good results.

We then employed a diffusion process with initial condition $S^{0}=W$ , and iteratively updated **S** by the following diffusion process:

$$S_{t+1}=\tau S_{t}\times P+\left(1-\tau \right)I_{N},$$ (2)

where **P** is a localized transition matrix of **W** and τ is a regularization parameter. Here, *K* nearest neighbor (KNN) is used to measure the local affinities. The localized row-normalized **P** is defined as

$$P\left(i,j\right)=\frac{W\left(i,j\right)}{\sum_{k\in knn\left(i\right)}W\left(i,K\right)}\delta \left\{j\in knn\left(i\right)\right\}.$$ (3)

This local similarity measurement is based on the metric learning (Wang et al., 2012). In single-cell clustering, this definition makes similarities between non-neighboring cells to zero and encodes the similarity to the *K* most similar cells for each cell with the assumption that local neighbors are more reliable than remote ones. For the following clustering analysis, we used spectral clustering (Park and Zhao, 2018) to assign labels to cells on the diffusion graph since it has the advantage of capturing the global structure of the graph.

### Estimating the Optimal Clustering Number

Clustering algorithms always suffer the limitation of selecting an optimal number of clusters. Here, we applied a separation cost method to estimate the optimal number of clusters based on the diffused graph (Zelnik-manor and Perona, 2005). The separation cost analyzes the eigenvectors of the affinity matrix and aims to find the optimal cluster number by minimizing the cost function. Given several communities C, the method aims at finding an indicator matrix Z(R)=XR , $Z\in R{,}^{n\times C}$ where **X** is the matrix of top eigenvectors of the affinity Laplacian, and **R** is a rotation matrix. Let

$$\left[M{\left(R\right)}_{i}\right]=\max{\left[Z\left(R\right)\right]}_{i,j}.$$ (4)

Defining the following cost function to be minimized:

$$J\left(R\right)=\sum_{i=1}^{n}\sum_{j=1}^{C}\frac{{\left[Z\left(R\right)\right]}_{i,j}^{2}}{{\left[M\left(R\right)\right]}_{i}^{2}}.$$ (5)

The optimal number of clusters is the one of communities that result in the largest drop in the value of *J*(R).

## Results

### Simulation Evaluation of LSSD

In the self-diffusion iteration process, the steps t need to be set properly. Too much diffusion may result in over-smoothed information for a given graph. Here, we conducted a simulation experiment to investigate the selection of iteration steps, meanwhile evaluating the clustering performance of LSSD in scRNA-seq clustering. We applied R package “Splatter” (Zappia et al., 2017) to simulate scRNA-seq read count data with 2,000 cells, 10,000 genes, and 10 groups. Normalized mutual information (NMI) (Zhang, 2015) and adjusted Rand index (Hoffman et al., 2015) were used as a measurement of consistency between the obtained partitions and the ground truth. NMI and ARI range between 0 and 1, where a higher value indicates higher concordance. We first computed the local scaling affinity on the simulation scRNA-seq. The self-diffusion process was then iteratively performed on the affinity matrix with the diffusion iteration steps varying t from 2 to 15. We applied spectral clustering which is a graph-based clustering method to obtain cell labels.

**FIGURE 2:**
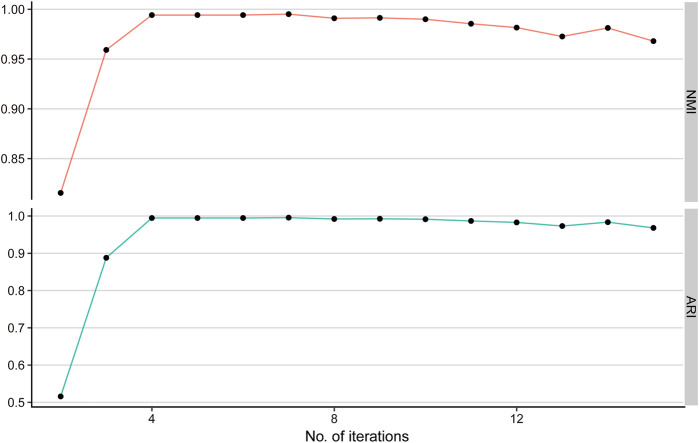
Simulation study for selecting the best iteration step. NMI and ARI values for varying iteration steps from 2 to 15 determine the optimal iteration step t = 4.

The result indicates that the NMI and ARI achieve the highest value when the iteration step t = 4(NMI = 0.99, ARI = 0.99) (Figure 2). Therefore, we selected iteration t =4 for the downstream analysis.

**FIGURE 3:**
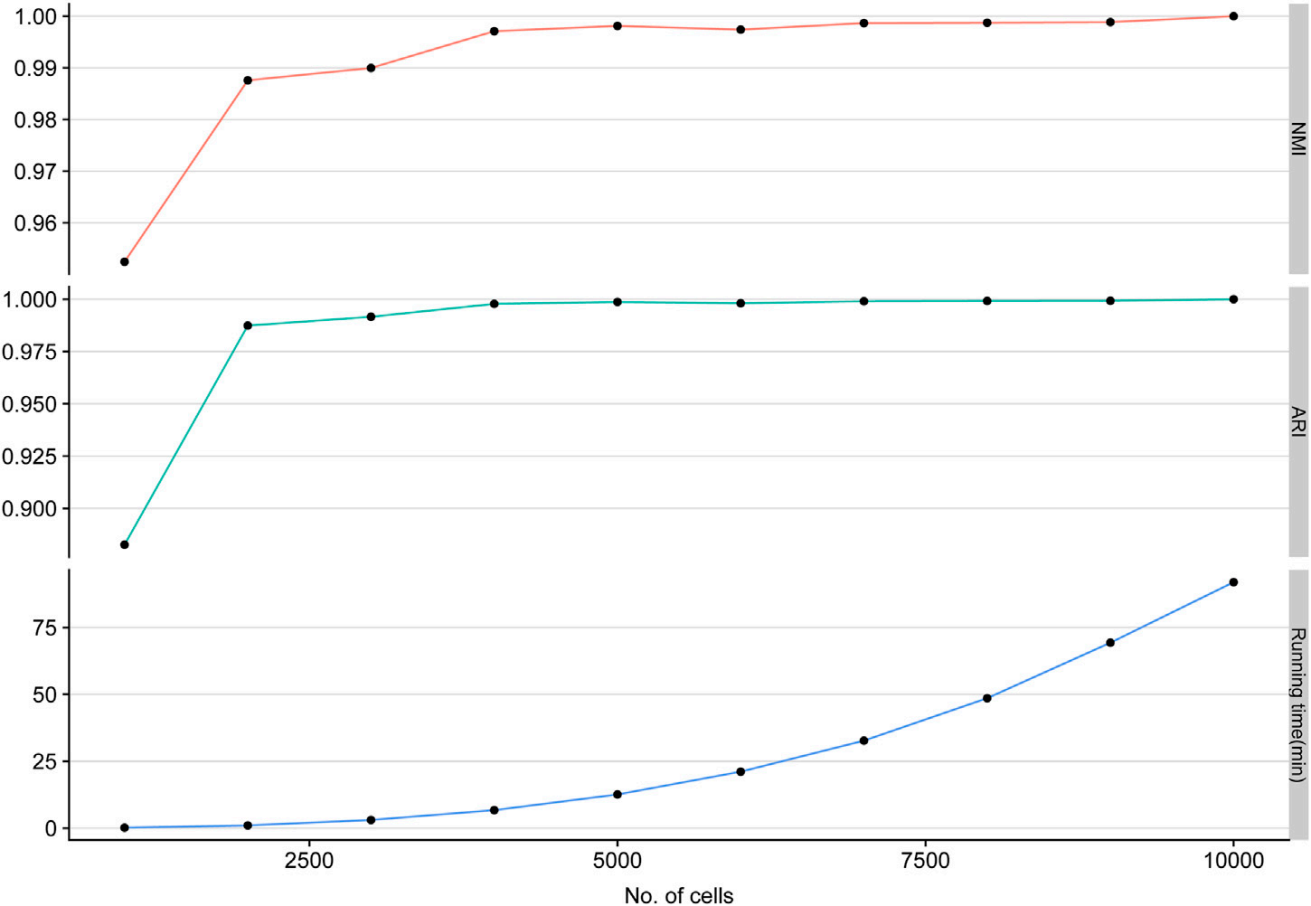
Applying LSSD on various sample sizes of simulated cells. LSSD applied on different sample sizes indicates the high stability of clustering performance measured by NMI, ARI, and running time.

Single-cell clustering methods are always confronted with the increasing number of scRNA-seq cells. To evaluate the scalability of LSSD, we performed scRNA-seq clustering on various sample sizes varying from 1,000 to 10,000 cells with 10,000 genes and 10 groups. We find that NMI and ARI values are tending toward stability (NMI, ARI close to 1), and the running times of LSSD scale linearly with the increasing number of cells. These results demonstrate that our LSSD approach is very robust to cell size variation. The running time increases with the growth of the cell sample size, indicating that LSSD can be an effective modeling for the analysis of large datasets (Figure 3).

### Case Study on Real scRNA-Seq Data

**FIGURE 4:**
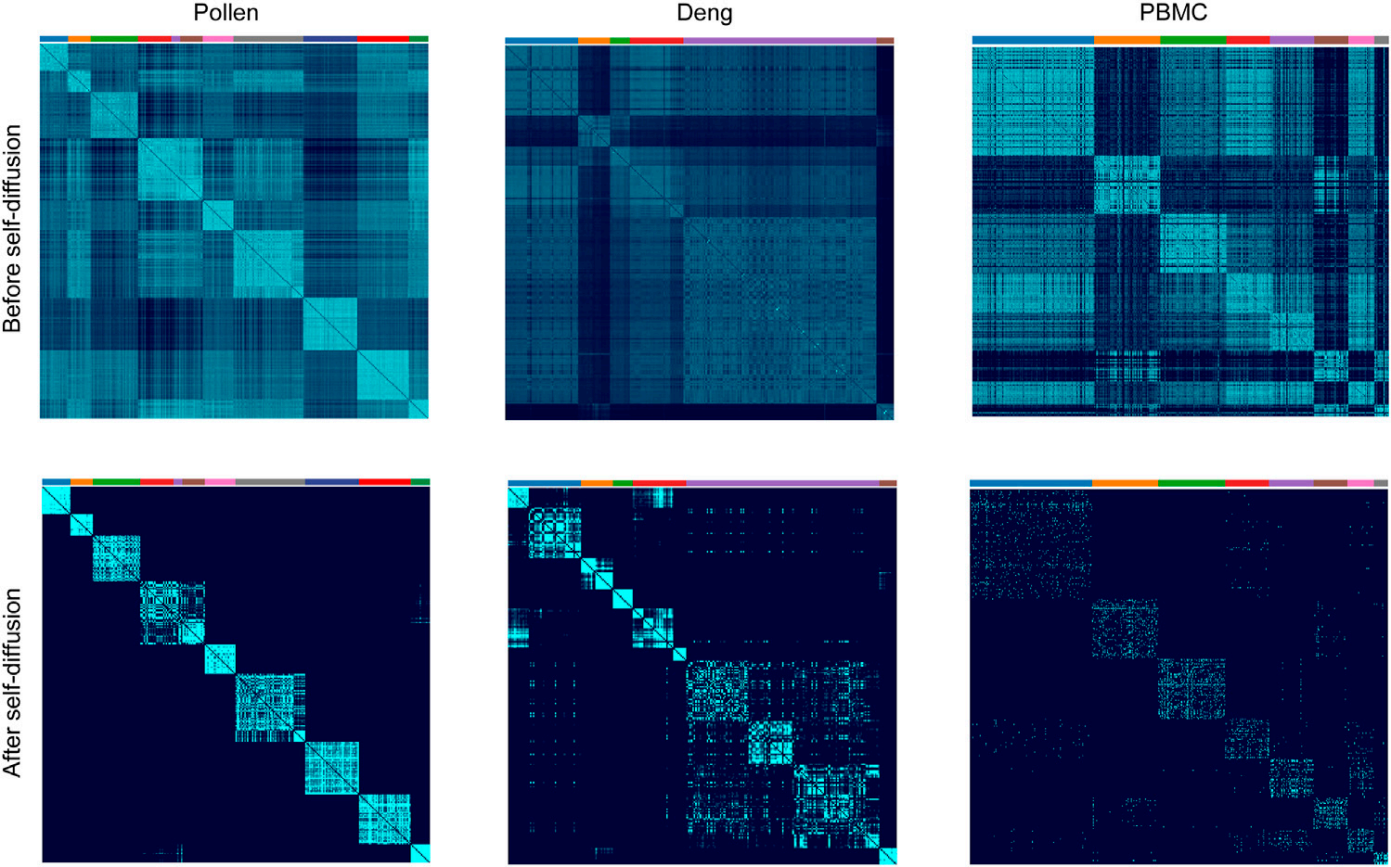
Cell similarities’ comparison of before and after self-diffusion on Pollen, Deng, and PBMC datasets. Visual inspection of the cell’s similarities after self-diffusion using LSSD reveals an enhancement of intercluster connection.

To further evaluate the performance of our LSSD approach, we applied it on three real scRNA-seq datasets. The Pollen dataset has 301 cells and 23,730 features, including 11 cell populations from neural cells and blood cells. The Deng dataset has 268 cells and 22,431 features with six cell populations. The 10X PBMC dataset contains 4271 cells and 16,449 features with eight groups from the peripheral blood mononuclear cells. To better show the effectiveness of the LSSD approach for cell-to-cell similarities’ denoising, we did not carry out gene filtering on the datasets. The local scaling affinity was first constructed using the scRNA-seq gene expression matrix. We then implemented self-diffusion on the affinity with iterations = 4. To investigate the ability of the self-diffusion process in network denoising, we compared the cells’ similarities before and after the diffusion process. Visual inspection of cell similarities after self-diffusion reveals a clear enhancement of edges within each cluster (Figure 4). The similarity improvement is particularly obvious for the Pollen dataset. The reason is that the self-diffusion process enables weak similarities connected by low-weight edges to disappear, contributing to reducing the noise and facilitating the strong similarities connected by high-weight edges. Since the cell-to-cell similarities are largely enhanced, the diffused graph becomes an appealing input for accurate detection of clusters.

**FIGURE 5:**
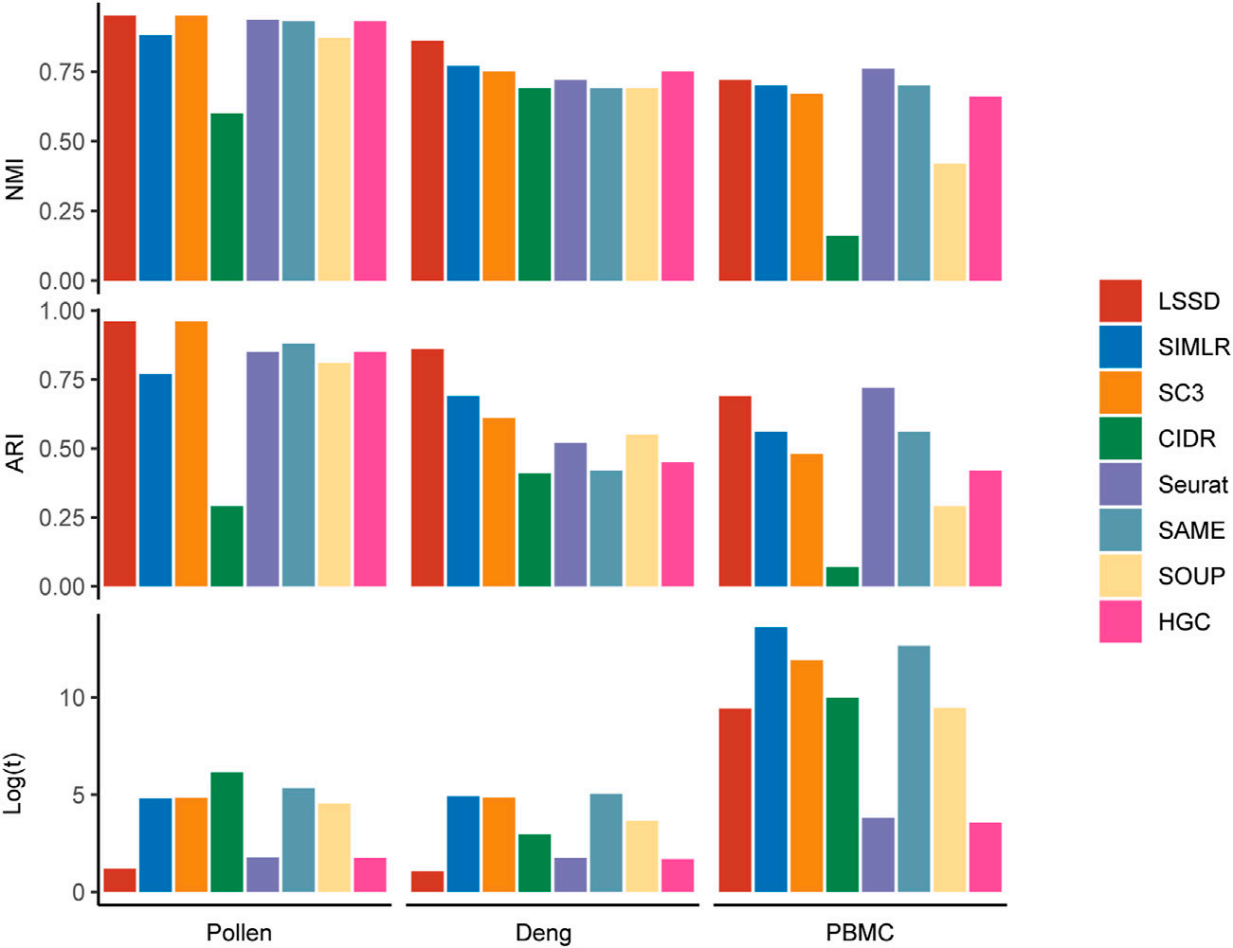
Comparison of clustering performance using LSSD, SIMLR, SC3, CIDR, Seurat, SAME, SOUP, and HGC. The benchmark results on the three real scRNA-seq datasets are evaluated by NMI and ARI, where the larger values indicate more concordance between the clustering labels and the true labels. Less running time means higher computational efficiency. Our LSSD approach shows appealing clustering performance compared with the other seven methods.

To extensively demonstrate the clustering effectiveness of LSSD on the three real datasets, we performed comparison of LSSD with seven other scRNA-seq clustering methods, including SIMLR (Wang et al., 2017), SC3 (Kiselev et al., 2017), CIDR (Lin et al., 2017), Seurat (Hao et al., 2021), HGC (Zou et al., 2021), SOUP (Zhu et al., 2019), and SAME clustering (Huh et al., 2020) (Figure 5). We used the NMI and ARI values to measure the consistency between obtained clustering labels and the ground truth. Running time was applied to compare the computational efficiency. On the Pollen dataset, the NMI and ARI of LSSD are 0.95 and 0.96, respectively. Although NMI and ARI values derived from SC3 and LSSD are the same, the running time of LSSD is much less than that of SC3. On the Deng dataset, the NMI and ARI of the LSSD method are 0.86 and 0.86, respectively, showing much better consistency than the other seven methods. The running time of LSSD on Pollen and Deng datasets is also far more efficient than that of other methods. These results indicate that our LSSD consistently outperforms the other seven methods on the Pollen and Deng datasets, especially on the Deng dataset where the cell-similarity pattern is difficult to discern. On the larger PBMC dataset, our approach also achieves appealing performance except for the Seurat method.

**FIGURE 6:**
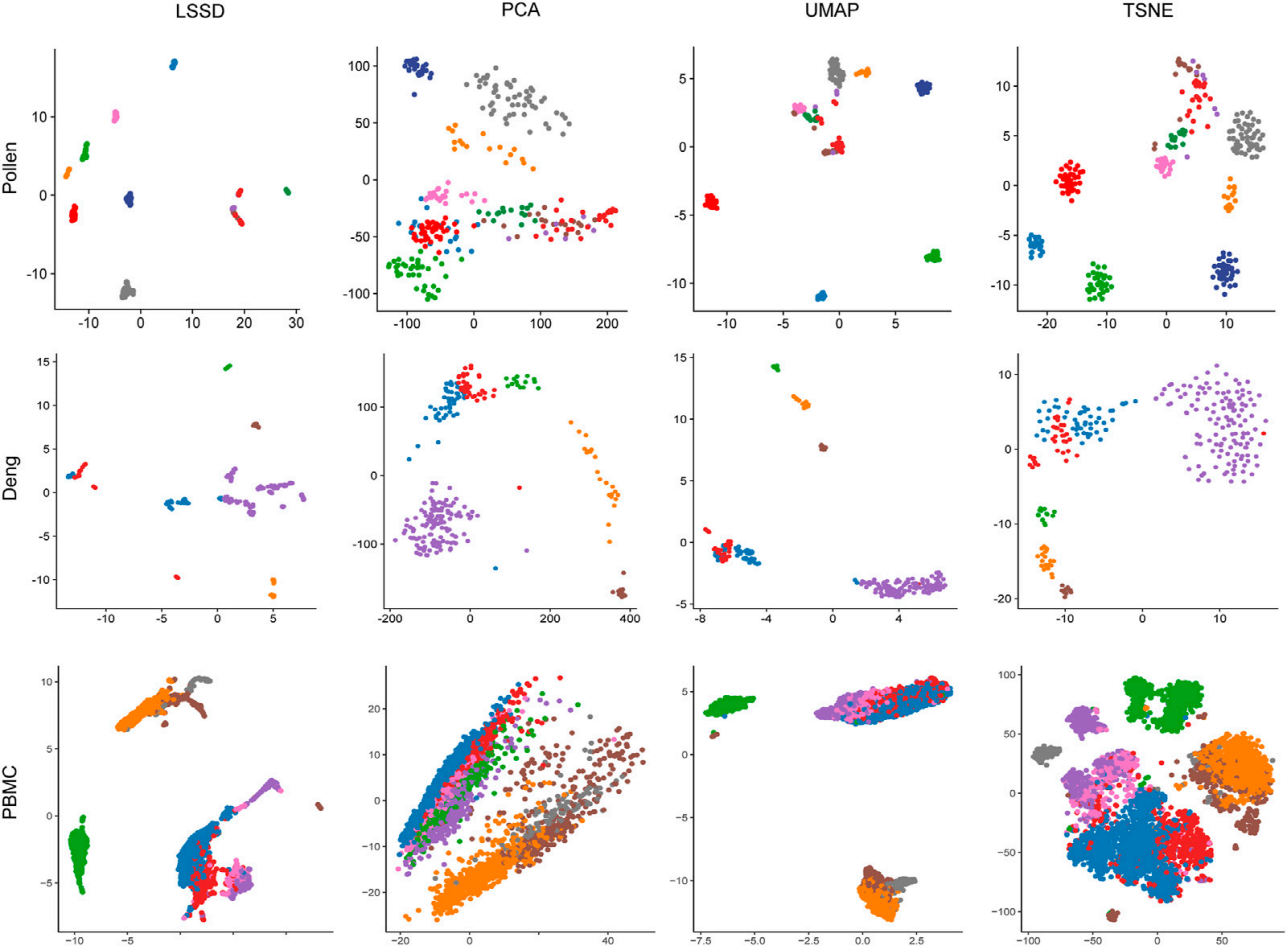
Comparison of 2D visualization using different dimension reduction methods on Pollen, Deng, and PBMC datasets. The diffused graph incorporating the UMAP method reveals more cohesion cluster representation. Each color represents a cell population with true labels.

To evaluate the visualization effectiveness, we used the diffused graph incorporating UMAP (Armstrong et al., 2021) to visualize the cell populations of the three real datasets. Each cell population was colored with true labels. Benchmarking against three other dimensionality reduction methods, cell samples after using the LSSD approach are much more tightly distributed in the two-dimensional space (Figure 6).

**FIGURE 7:**
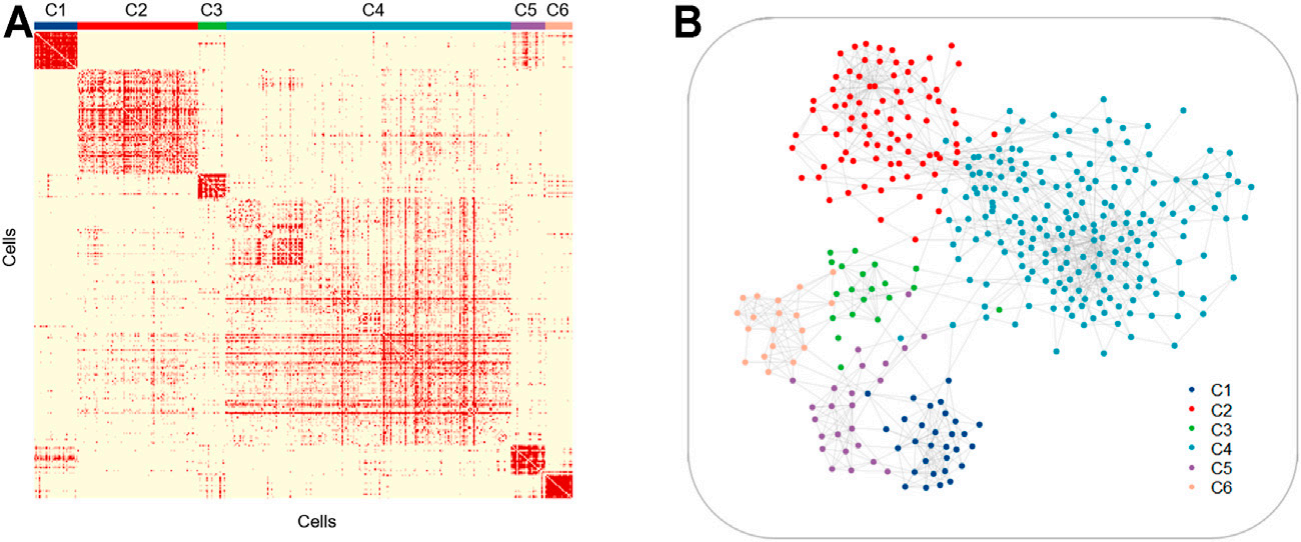
Similarities of the six colorectal tumor cell clusters. Similarity matrix **(A)** and cell network **(B)** for 375 colorectal tumor cells illustrating the tightness of connectivity within clusters.

**FIGURE 8:**
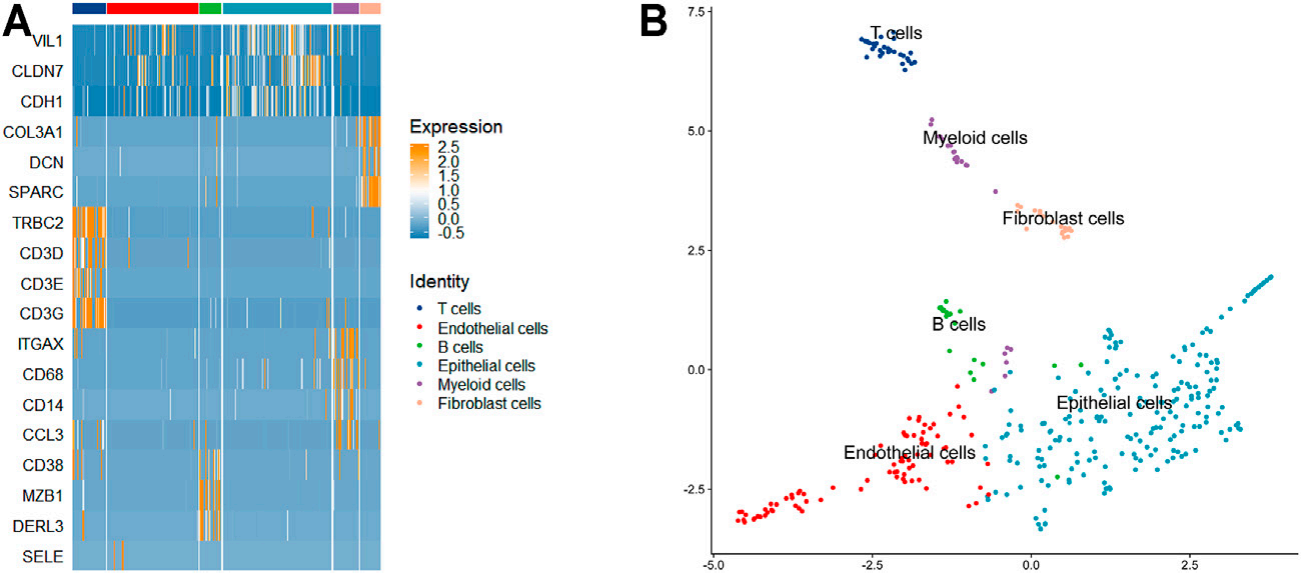
Cell cluster annotation using known gene markers. **(A)** Heatmap of several gene markers in the six cell identities. **(B)** 2D visualization of the identified cell types using UMAP.

Intratumor heterogeneity exists among tumor cells. Currently, the single-cell sequencing technology has been applied widely in various fields, but the most common application is in tumor research (Patel et al., 2014) (Giustacchini et al., 2017). The study of tumor cells using the single-cell technique has greatly promoted the understanding of intratumor heterogeneity and the development of antitumor therapeutic strategies. To explore the ability of LSSD in dissecting tumor cellular heterogeneity, we applied LSSD to 375 colorectal tumor cells assembled by Li et al. (2017). Since many genes are not informative, we first filtered out invariable genes and chose highly variable genes by the variance-to-mean ratio (Stuart et al., 2019). After preprocessing, 200 genes were kept for downstream analysis. We then constructed the local scaling affinity to measure the cell similarities on the preprocessed samples. Self-diffusion was performed on the local scaling affinity to further enhance the cell similarities with iteration step *t* = 4. The diffused graph enables effective clustering with six clusters estimated by separation cost methods (Supplementary Figure S1). Visual inspection illustrates that the diffused graph presents six clear cluster patterns corresponding to different cell groups (Figure 7A). The network visualization also indicates strong intercluster similarity, shown by the tightness of connectivity within the same subgraph and relatively few connections in between (Figure 7B). To annotate the cell clusters with meaningful biological types, we applied known gene markers to define cell types. These gene makers include TRBC2, SELE, CD38, VIL1, ITGAX, and SPARC. We compared their expression probability distribution in each cluster by a violin plot. The gene makers TRBC2, SELE, CD38, VIL1, ITGAX, and SPARC present a higher expression level in C1, C2, C3, C4, C5, and C6, respectively (Supplementary Figure S2). Therefore, the six clusters (C1–C6) of the colorectal tumor cells were annotated as T cells, endothelial cells, B cells, epithelial cells, myeloid cells, and fibroblasts cells with significantly differential gene marker expression (Figure 8A). The annotated cell-identity clusters were then visualized using a LSSD + UMAP representation in two-dimensional space (Figure 8B). Finally, we performed gene set enrichment analysis based on differentially expressed genes derived from each cell cluster (Supplementary Table S1). C1 (T cells), C3 (B cells), and C5 (bone marrow cells) are significantly correlated with immune pathways. Cancer-associated fibroblasts (CAFs) are one of the major cytokines which are responsible for the structure-related changes of extracellular matrix during tumorigenesis (Malik et al., 2015). C6 (fibroblast cells) is associated with the extracellular matrix pathway.

### LSSD Can Improve the Performance of Network Fusion

To comprehensively prove the network enhancement of our LSSD, we applied it on network fusion for cancer subtyping. Three data types including mRNA expression, DNA methylation, and miRNA for 105 breast cancer patients were used. We replaced the network construction in similarity network fusion (SNF) (Wang et al., 2014) with an LSSD map in every data type and then performed the network fusion process. We compared their performance using survival analyses with the log-rank test using the “survival” package. Overall survival (OS) was employed to explore the association with identified subtypes. The subtypes identified using network fusion with our LSSD map showed a much more significant association with OS (Supplementary Figure S3A, *p* = 3.75E-12, log-rank test) than the counterpart based on the original SNF method (Supplementary Figure S3B, *p* = 4.1E-5, log-rank test). The reason is that the local scaling affinity can make balance in the data scale difference, and self-diffusion process further enhances the network learning, while parameter setting in SNF is ambiguous and sensitive to data scale.

## Discussion

Single-cell RNA sequencing has enabled gene transcriptomic profiling to be studied at the individual cell level, advancing our understanding of the cellular heterogeneity and underlying mechanisms (Buettner et al., 2015; Kaur et al., 2019). Clustering scRNA-seq data into different cell types has the potential to characterize multicellular organisms and reveal unknown heterogeneity. This methodology explores cellular heterogeneity at an unprecedented resolution which differs from traditional bulk RNA-seq and microarray data, where gene expression measurements are averaged over thousands of cells from a sample. However, scRNA-seq data always contain numerous zero-value observations and redundant information. In this article, we propose local scaling self-diffusion (LSSD) modeling to enhance the cell similarity learning for unsupervised clustering analysis of scRNA-seq data. This similarity measurement can greatly improve the effectiveness of downstream clustering tasks, leading to accurate cell type identification. In the LSSD approach, local scaling affinity infers the self-turning of cell–cell distance, followed by the iterative self-diffusion process to denoise the network. LSSD has the advantage of eliminating weak similarity, reducing feature redundancy, and enhancing strong similarity along the diffusion of the network. In cell type identification, LSSD incorporating spectral clustering can overcome the limitation of high levels of cell noise and dropout events. The simulation study illustrated LSSD has strong robustness to the cell sample sizes, making it a scalable analytical framework for single-cell clustering. In addition, benchmarking against seven other single-cell clustering methods on three real datasets, LSSD showed higher NMI and ARI values, while requiring less computational complexity. Finally, to further evaluate the performance of LSSD, we carried out cell type identification on scRNA-seq of 375 colorectal tumor cells. Using known gene markers, we identified six cell types and analyzed the biological pathways associated with each cell type. Additionally, combining with the network fusion step, we found our local scaling self-diffusion map can largely improve the performance of the original SNF method in subtype identification.

## Conclusion

Due to the complexity in scRNA-seq data, there are still many computational challenges for analysis of these data. LSSD’s simplicity and efficiency make it an appealing unsupervised clustering approach for these challenges. As abundant scRNA-seq data become easier to obtain, we expect our LSSD model combining these data can give a more comprehensive view of disease and biological processes.

## Data Availability

Publicly available datasets were analyzed in this study. These data can be found here: Colorectal tumors cells and Deng dataset can be found at Gene Expression Omnibus (https://www.ncbi.nlm.nih.gov/geo/) under accession numbers GSE81861 and GSE45719, respectively. Pollen dataset can be found at (http://www.ncbi.nlm.nih.gov/Traces/sra/) under accession number SRP041736. The 10X PBMC dataset was downloaded from website (https://support.10xgenomics.com/single-cell-gene-expression/datasets/2.1.0/pbmc4k). The R package for LSSD implementation is available at: https://github.com/DuanX8/LSSD.
