# Supplementary material for: Dissecting Cellular Heterogeneity Based on Network Denoising of scRNA-seq Using Local Scaling Self-Diffusion
Source: Front Genet. 2022 Jan 10;12:811043. doi: 10.3389/fgene.2021.811043 (PMC8784844; doi:10.3389/fgene.2021.811043)
Supplement: Supplementary file 1 [file DataSheet1.docx]

## **Supplementary material**


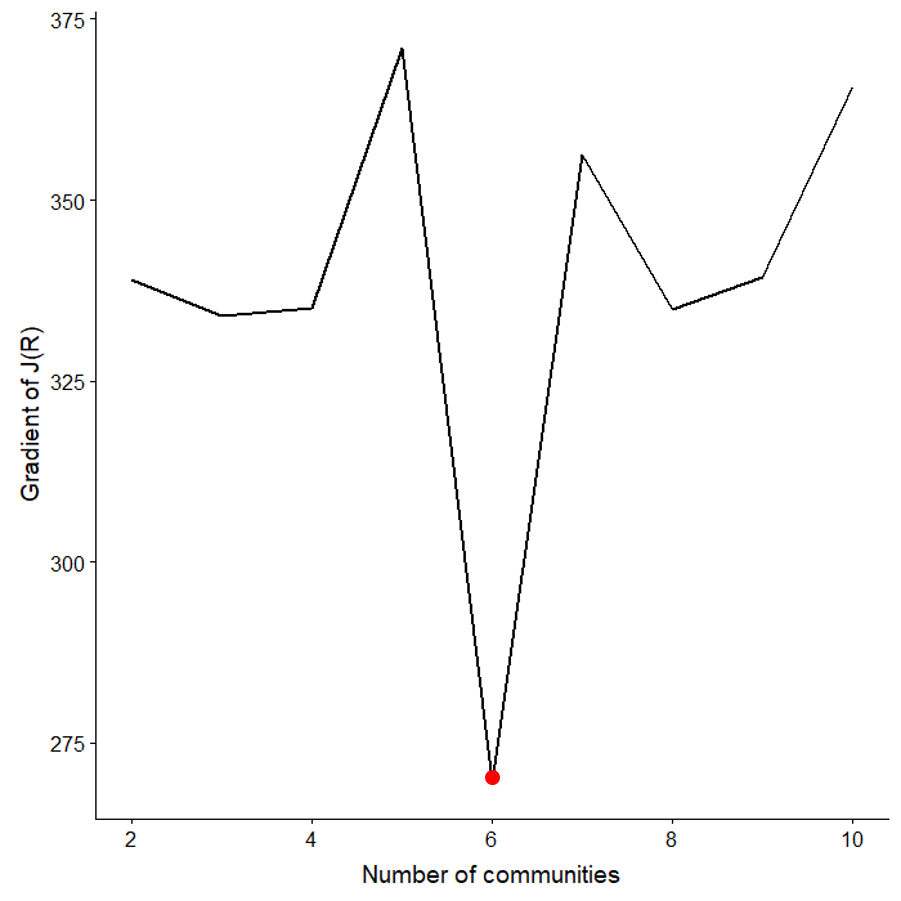


**Figure S1.** **The optimal clustering number is C=6 estimated by separation cost which minimizes the gradient of J(R) (red point).**

**
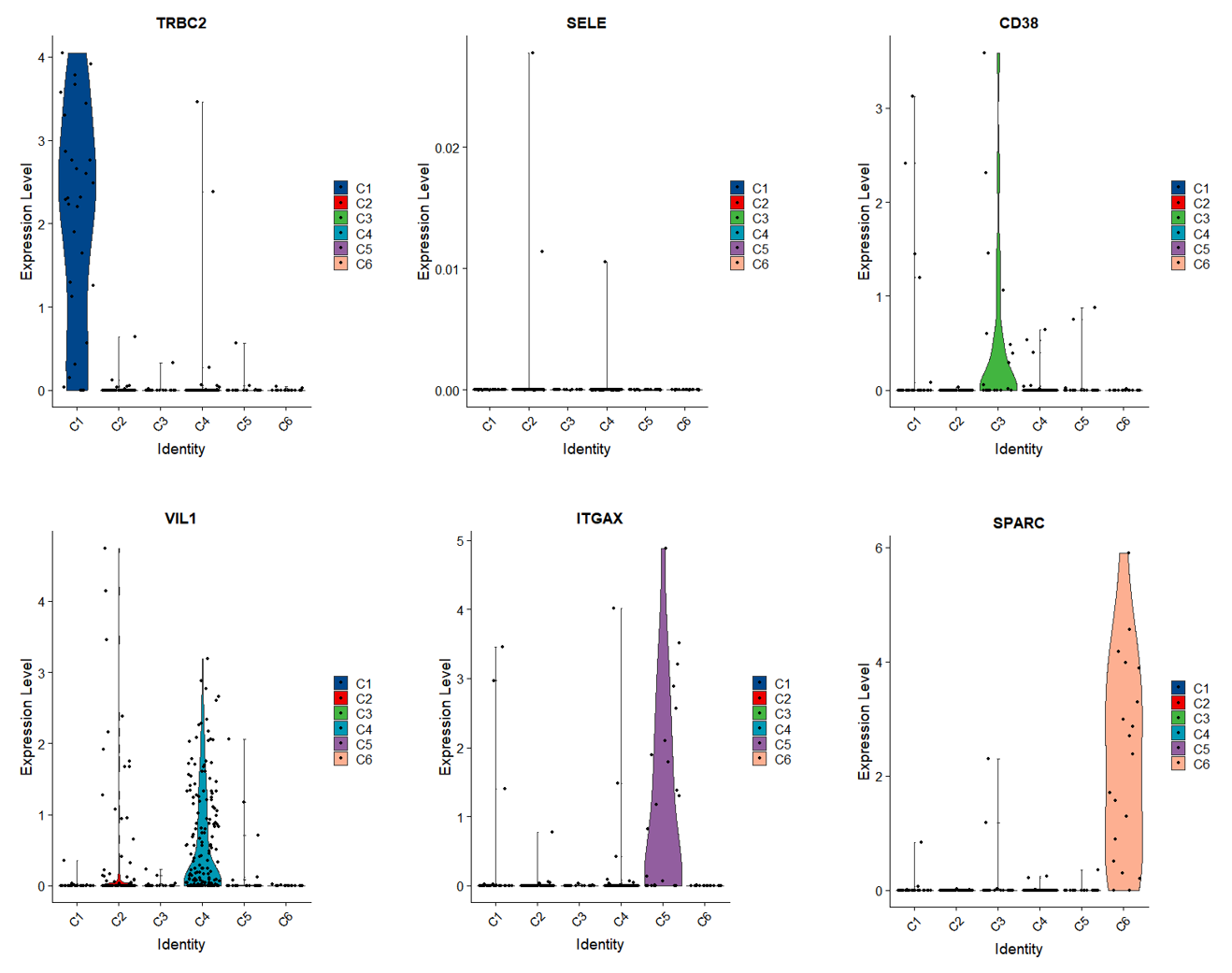
**

**Figure S2. Violin plot showing expression probability distributions of known gene markers in each cluster.**


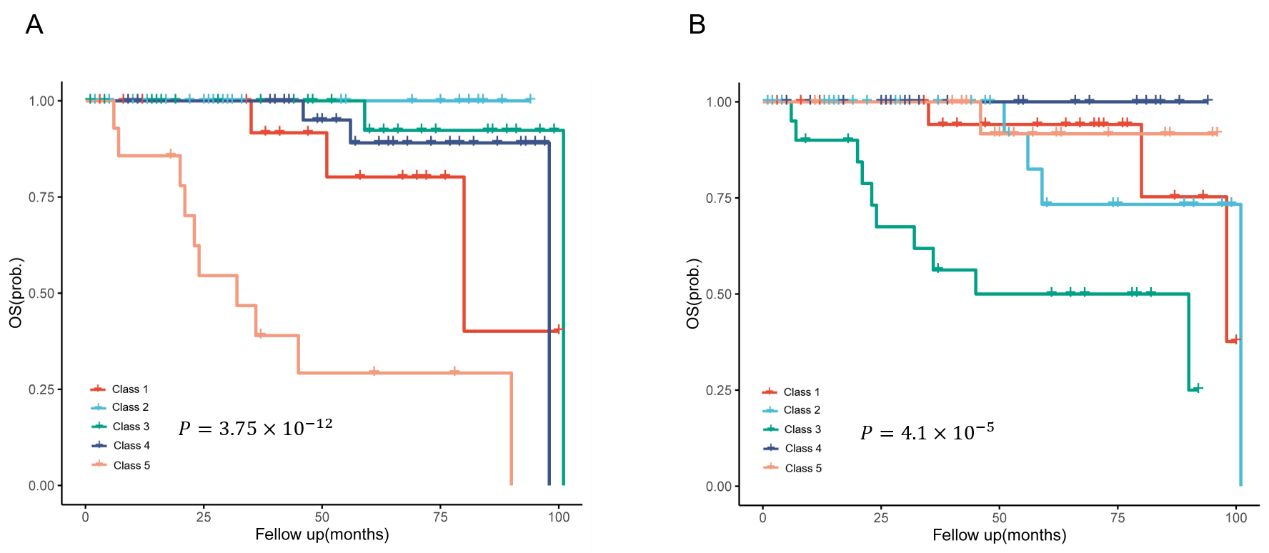


**Figure S3: Comparison performance of breast subtyping between network fusion with local scaling self-diffusion (A) and the original SNF methods (B).**

**Table S1. Pathway analysis of the six colorectal tumor cell types**

|  | Gene list | Pathway | *P* value |
| --- | --- | --- | --- |
| C1 | \| CD2 \| CD52 \| \| --- \| --- \| \| TRAC \| CD3D \| \| CD3E \| CD247 \| \| TRBC2 \| PTPRC \| \| GZMA \| CD69 \| | T cell receptor signaling pathway  T cell receptor complex  PD-1 signaling  T cell differentiation  T cell differentiation in thymus | 8.30E-06  1.13E-05  3.56E-05  4.05E-4  5.8E-3 |
| C2 | \| MT-CO3 MT-CO2 \| \| --- \| \| RP5-857K21.7 MTRNR2L8 \| \| MTND2P28 MT-ND4 \| \| RP5-857K21.11 \| | Ventricular preexcitation  Mitochondrial myopathy  Oxidative phosphorylation  Ventricular arrhythmia  Thermogenesis | 3.68E-07  6.72E-07  5.27E-06  6.96E-05  4.72E-05 |
| C3 | \| MZB1 \| CPNE5 \| \| --- \| --- \| \| DERL3 \| IGHA2 \| \| IGLL5 \| SLAMF7 \| \| CD27 \| TNFRSF17 \| \| IGLC1 \| TMEM156 \| | \| regulation of B cell activation \| \| --- \| \| adaptive immune response \| \| B cell activation \| \| lymphocyte activation \| \| TNFs bind their physiological receptors \| \|  \| | \| 0.00109 \| \| --- \| \| 0.00498 \| \| 0.00942 \| \| 0.0104 \| \| 0.0115 \| \|  \| |
| C4 | \| TSPAN8 \| CD9 \| \| --- \| --- \| \| SLC1A2 \| CDH1 \| \| HNF4A \| ELF3 \| \| MGST1 \| MUC13 \| \| CEACAM5 \| SOX9 \| | \| apical part of cell \| \| --- \| \| Gonadal neoplasm \| \| Dysgerminoma \| \| Genital neoplasm \| \| Germinoma \| \|  \| | \| 0.000975 \| \| --- \| \| 0.00524 \| \| 0.00606 \| \| 0.0104 \| \| 0.0197 \| |
| C5 | \| MS4A7 \| MRC1L1 \| \| --- \| --- \| \| STAB1 \| HLADQA2 \| \| MRC1 \| CD300E \| \| AIF1 \| HLADQA1 \| \| MS4A6A \| TYROBP \| | \| innate immune response \| \| --- \| \| cellular response to interferon-gamma \| \| Phagosome \| \| defense response \| \| response to interferon-gamma \| | \| 0.0012 \| \| --- \| \| 0.00123 \| \| 0.00159 \| \| 0.00172 \| \| 0.0019 \| \|  \| |
| C6 | \| BGN \| COL15A1 \| \| --- \| --- \| \| COL4A1 \| SPARCL1 \| \| SPARC \| FBN1 \| \| COX7A1 \| AEBP1 \| \| CALD1 \| C11orf96 \| | \| collagen-containing extracellular matrix \| \| --- \| \| extracellular matrix \| \| extracellular matrix structural constituent \| \| Extracellular matrix organization \| \| basement membrane \| \| extracellular matrix component \| \| extracellular matrix binding \| | \| 3.09E-09 \| \| --- \| \| 5.63E-08 \| \| 2.21E-06 \| \| 6.53E-05 \| \| 9.90E-05 \| \| 0.00188 \| \| 0.00272 \| |
